# The ERGtools2 package: a toolset for processing and analysing visual electrophysiology data

**DOI:** 10.1007/s10633-025-10017-2

**Published:** 2025-04-12

**Authors:** Moritz Lindner

**Affiliations:** 1 Department of Neurophysiology, Institute of Physiology and Pathophysiology, Philipps-University Marburg, 35037 Marburg, Germany; 2 The Nuffield Laboratory of Ophthalmology, Sleep and Circadian Neuroscience Institute, Nuffield Department of Clinical Neurosciences, University of Oxford, Oxford, UK; 3 Department of Ophthalmology, Philipps University Marburg, 35037 Marburg, Germany

**Keywords:** Visual electrophysiology, R package, Data analysis, Open source, Data preservation

## Abstract

**Purpose:**

To introduce ERGtools2, an open-source R package for processing, analysing and long-term storing visual electrophysiology data.

**Methods:**

A dataset comprising Electroretinogram (ERG) recordings of C57Bl/6J mice, subjected to standard ISCEV stimuli, was used to present the functionality of ERGtools2. ERGtools2 stores and organizes all recordings, metadata, and measurement information from an individual examination in a single object, maintaining raw data throughout the analysis process.

**Results:**

A standard workflow is presented exemplifying how ERGtools2 can be used to efficiently import, pre-process and analyse ERG data. Following this workflow, basic ERG measurements and visualisation of a single exam as well as group statistics are obtained. Moreover, special use cases are described, including for the handling of noisy data and the storage of data in the HDF5 format to ensure long-term preservation and accessibility.

**Conclusions:**

ERGtools2 provides a comprehensive, flexible, and device-independent solution for visual electrophysiology data analysis. Its emphasis on maintaining raw data integrity, combined with advanced processing and analysis capabilities, makes it a useful tool for preclinical and clinical research applications. The open-source nature and the use of open data formats promote reproducibility and data sharing in visual neurosciences.

**Supplementary Information:**

The online version contains supplementary material available at 10.1007/s10633-025-10017-2.

## Introduction

Visual electrophysiology techniques, including electroretinography (ERG) and visually evoked potentials (VEP) enable a direct functional assessment of the visual pathway, from photoreceptors to the visual cortex [1–5].

Emerging applications, such as using electrophysiology as an objective endpoint in vision prosthetics, require flexible analysis strategies. For instance, electrophysiological responses rescued or restored by a novel therapeutic approach might be too weak to be reliably measured using standard ERG/VEP readouts while still containing important evidence of the efficacy of that approach. In such cases, alternative analysis strategies like performing discrete Fourier transformations on Flicker ERGs may provide higher sensitivity [6].

Moreover, measures to ensure reusability and long-term preservation of data are increasingly required by research funding bodies, clinical regulators, and animal welfare authorities. These considerations are reflected in the FAIR (“Findable, Accessible, Interoperable and Reusable”) Data Principles [7]. Despite recent efforts [8], in visual electrophysiology a standard data format in line with the FAIR principles is yet not established.

Here we present ERGtools2, an open-source framework for processing and analysing retinal electrophysiology data in R [9]. It comes with command-line tools as well as a visual interface and offers data storing in an open, self-documenting format. In this paper, technical aspects of the ERGtools2 package are described in Methods and examples of its functionality are presented in Results. ERGtools2, in its version 0.85, is available as supplemental material (Supplemental file 1) to this paper and the latest version can be downloaded from GitHub (https://github.com/moritzlindner/ERGtools2). Example code is provided in two supplementary files (Supplemental file 2 and 3).

## Material and methods

ERGtools2 was developed in R, version 4.3.1, a free language for statistical computing and RStudio 2023.06.1 IDE on a Linux system (Ubuntu 22.04 LTS). The package was additionally tested on Windows 10.

The core of the ERGtools2 package is the ERGExam object, which stores all recordings from an individual examination. All data in an ERGExam object is kept as raw data and functions for filtering and averaging the data or rejecting poor quality/outlier trials are stored within the object. Moreover, a log is kept of all changes made to the data by the user. A detailed description of the ERGExam object can be found in the Supplementary Methods File. A description of all methods to access or modify the object is found in the package’s help pages accessible by typing “*?ERGtools2::`ERGtools2-package`”*. Key commands are additionally listed in Table 1.

**Table 1:** Important functions and methods implemented in the ERGtools2 package

Command	Description
newERGExam()	Create a new ERGExam object
Save(), Load()	Saves and loads the ERGExam object into and hdf5 file
SetStandardFunctions()	Sets standard functions for processing ERGExam data, defining default functions for averaging, filtering, and signal rejection based on the stimulus type
AutoPlaceMarkers()	Automatically sets markers depending on the channel (e.g., ERG, VEP, OP) and stimulus type (Flash, Flicker)
Where()	Returns the indices of recordings matching the given criteria
Subset()	Subsets an ERGExam object into a new object of the same class
Measurements()	Returns the Measurements table
interactiveMeasurements()	Allows for interactive visual placement of markers
exploreERGExam()	Explores the ERGExam object interactively
ggERGExam()	Uses ggplot2 to plot the content of an ERGExam object
ggIntensitySequence()	Uses ggplot2 to plot a stimulus-response sequence for a list of ERG exams

Animal work and data acquisition procedures relevant for the generation of the sample dataset used herein are also described in the Supplementary Methods File.

## Results

### A standard workflow

**Fig. 1:**
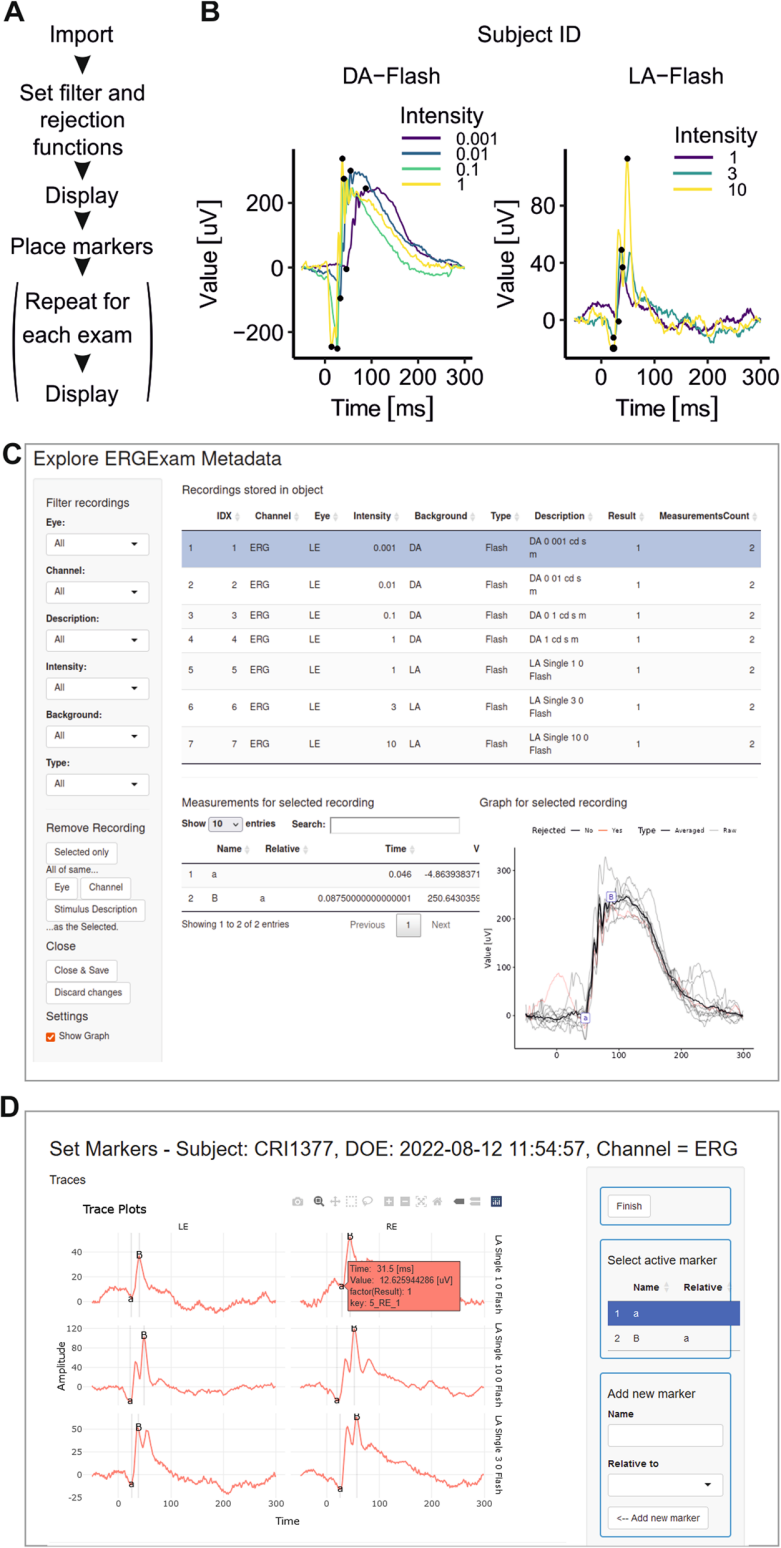
Visualization and manipulation of an ERGExam object using ERGtools2. **A** Standard workflow to import and process visual electrophysiology data using ERGtools2. **B** Visualization of an ERG exam from a left eye of a mouse as obtained by running *ggERGExam()*. Recordings are automatically colour coded by stimulus strength (‘intensity’ refers to the time-integrated luminance of the flash stimuli) and panelled out by adaptation state (“DA” and “LA”). **C** An interactive interface for viewing the content of an ERGExam object can be called typing *exploreERGExam().* Besides viewing, it also allows subsetting an individual ERGExam object. **D** An interactive interface for placing trace markers can be called via *interactiveMeasurements()*

ERGtools2 can import visual electrophysiology data from any file format that can be read into R. After import, the user can set functions for filtering, rejection of outlier traces and averaging of repeated measurements (trials) before the recordings can be visualized or wave markers (e.g. for a or b wave) can be placed. These standard steps for data processing are summarized in Fig. 1 A and an example of the output of the inbuilt method for exam visualization is given in Fig. 1 B. Interactive user interfaces for visualizing individual recordings from one exam or for placing wave markers are also available (Fig. 1 C and D).

**Fig. 2:**
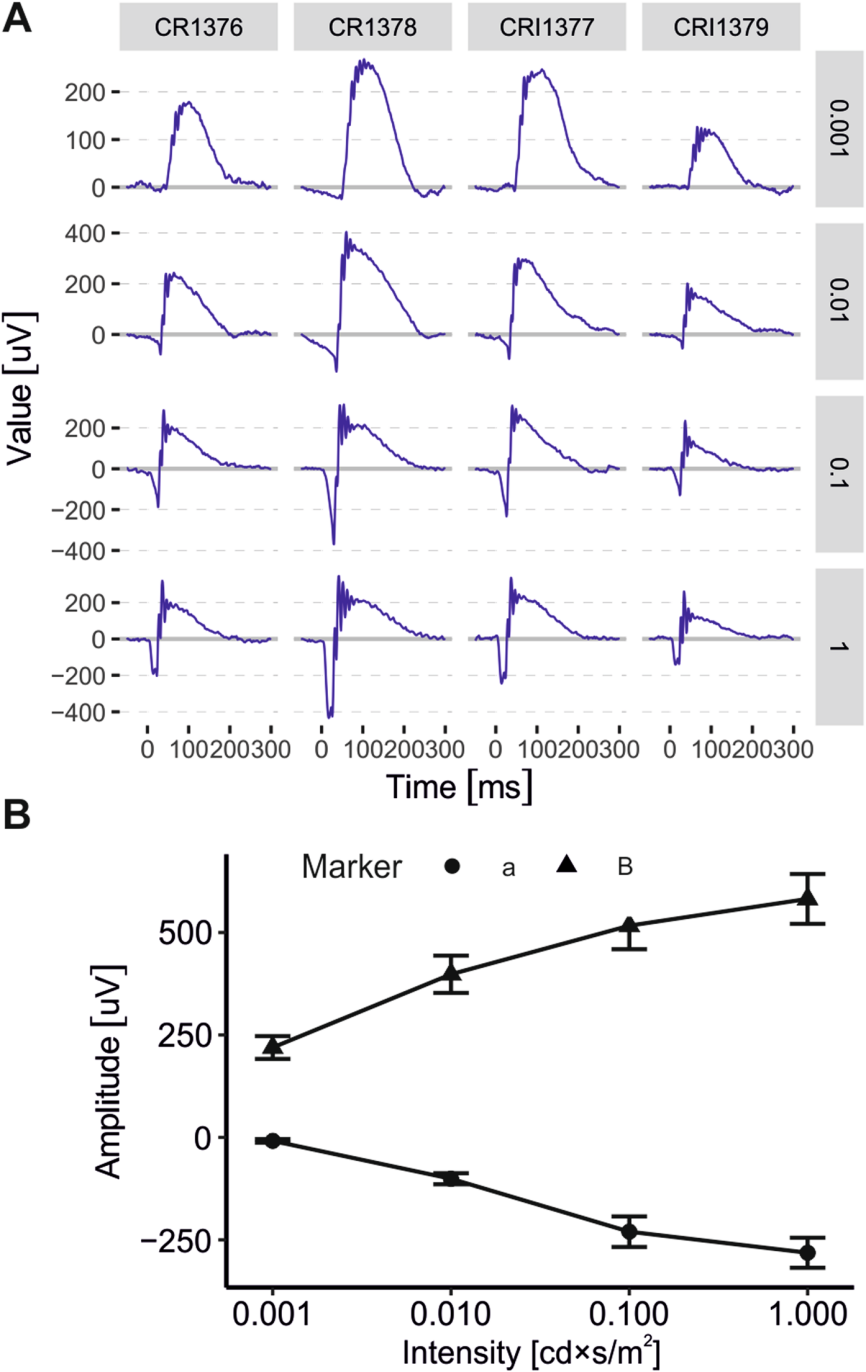
Visualization of multiple ERG exams. **A** Automatic visualization of a set of visual electrophysiology exams using *ggPlotRecordings()*. Individual traces are panelled out by subject (columns) and stimulus conditions (rows). Only recordings for dark-adapted stimuli are shown. **B** Summary stimulus-response curves for a and B-wave amplitudes from the exams shown in (A), visualized using *ggIntensitySequence()*

In a research setting, it may be necessary to compare exams acquired from multiple subjects often belonging to different experimental cohorts. To achieve this, ERGtools2 contains a set of functions to plot multiple exams side-by-side and to calculate or visualize group statistics (Fig. 2). A detailed description of the standard workflow is given in the Supplemental file 4.

### Special use cases

#### Automatic rejection of outlier recordings

**Fig. 3:**
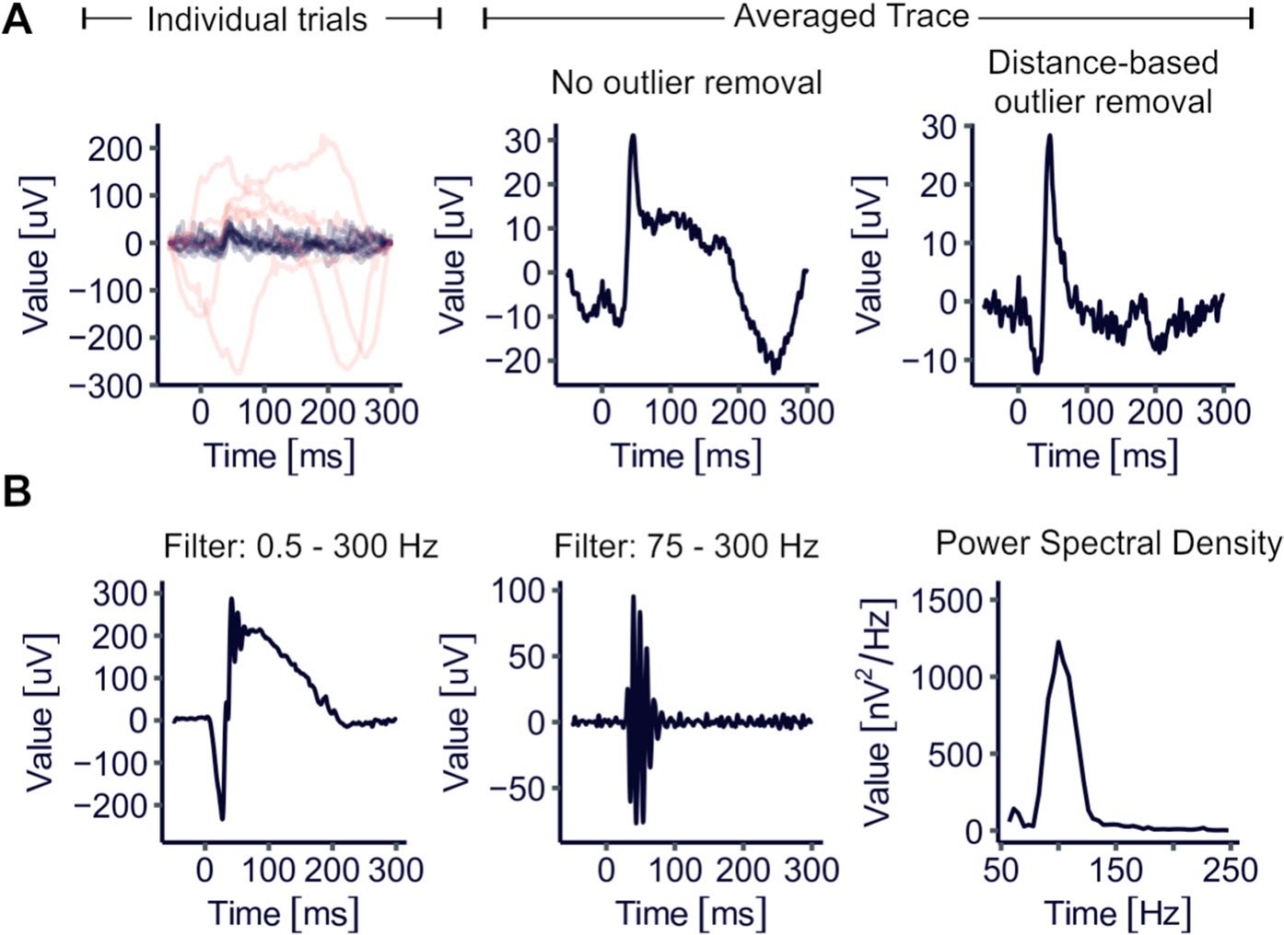
Special use cases: **A** Automatic rejection of outlier recordings using the *autoreject.by.distance()* function. Left panel: Raw trials of an ERG signal obtained in response to a light-adapted 1 cd*s/cm^2^ flash stimulus. Trials identified as outliers by *autoreject.by.distance()* are highlighted in red. Middle and right panel: Average traces from the raw trials shown on the left without (middle) and with (right) outlier trials removed. **B** Signal obtained in response to a dark-adapted 0.1 cd*s/cm^2^ flash stimulus. Left panel shows the recording after setting a 0.5–300 Hz bandpass filter using the *FilterFunction()* <—method and the 4th order Butterworth filter as implemented in the *filter.bandpass()* function. Middle panel, same recording, but with a 75–300 Hz bandpass filter in place, extracting the oscillatory potentials. Right panel: Identification of the predominant frequency components in the signal, calculating the spectral density by using the *PSD()* method

A particular challenge in processing visual electrophysiology data is dealing with occasionally poor signal-to-noise ratios. ERGtools2 offers functions that identify outlier trials based on their similarity to the other trials. The function *autoreject.by.distance()*, for instance, calculates a distance matrix between the trials and rejects those that deviate from all others by more than a threshold value, offering a user-independent, unbiased strategy for outlier removal (Fig. 3 A).

#### Extracting and analysing oscillatory potentials

Inner retinal network activity is reflected by the oscillatory potentials (OPs) which can be observed overlaying the B-wave in electroretinogram recordings [10]. In ERGtools2, the desired bandpass-filter window can easily be set for an individual recording using *FilterFunction()* method (Fig. 3 B, left and middle panel). In analogy, data can be Fourier-transformed to extract peak frequency and spectral power using the *PSD* (= “power spectral density”) method (Fig. 3 B, right panel).

#### Storing data for long-term preservation and shareability

To facilitate shareability and reusability of visual electrophysiology data ERGtools2 offers the option to store ERGExam objects in the Hierarchical Data Format version 5 (HDF5) [11] format using the *Save()* method. The HDF5 file created by *Save()* mirrors the structure of the ERGExam object. Exams once saved into an HDF5 file can be read into ERGtools2 again using *LoadERGExam()*.

## Discussion

ERGtools2 allows for efficient processing and analysis of visual electrophysiology data. It is written in the free open-source programming language R [9] and its code is itself free to use. ERGtools2 has an extensive command library allowing for complex and reproducible programming-based data processing and also offers an interactive interface which makes it possible to use the toolbox with minimal prior experience in programming. It supports storing data in the HDF5 format to support shareability, reusability and long-term preservation of visual electrophysiology data.

The concept of ERGtools2 is to store unfiltered raw data alongside with filters, as well as rejection and averaging functions. Those functions are then applied when viewing a recording or extracting measurements. This is particularly advantageous in research settings. For instance, when studying prosthetic visual restorative approaches like subretinal implants, optogenetic therapies or stem cell-based approaches [6, 12, 13] where electrical light responses may differ substantially from their physiological shapes and patterns, making post-hoc adjustment of filter functions necessary.

ERGtools2 is fully independent of the device and software used to acquire the data. Under the precondition that the system used for data acquisition supports raw data export, ERGtools2 can be used to process and analyse the data. Consequently, researchers can standardize their ERG data processing workflow, ensuring consistency and reliability regardless of the initial acquisition platform. This facilitates collaboration and data sharing across different laboratories or clinical sites. Notably, while ERGtools2 is written in R, embedding of the code into software written in other programming languages (e.g. into c# via RInside) is generally possible, facilitating the integration of ERGtools2 into existing workflows.

In line with the FAIR Data Principles [7], data once imported into ERGtools2 can be stored into HDF5 files [11], ensuring long-term accessibility. The structure in which ERGtools2 saves the data is self-documenting and resembles the structure of the ERGExam object. Thus, accessibility of the data is ensured also beyond the lifetime of any particular software, including ERGtools2 itself. Of note, there are ongoing efforts to implement data standards in visual electrophysiology like the ELVisML format based on XML (eXtensible Markup Language) [8]. A key advantage of the binary HDF5 format used by ERGtools2 lies in its ability to efficiently handle larger volumes of data.

In conclusion, ERGtools2 is an open-source data analysis platform with a particular focus on data integrity, shareability and long-term preservation. It enables both, efficient routine data analysis, achievable without prior experience in programming, as well as sophisticated customized data analysis and processing.

## Supplementary Information

Below is the link to the electronic supplementary material.

Supplementary file1 (ZIP 622 KB)

Supplementary file2 (PDF 392 KB)

Supplementary file3 (PDF 710 KB)

Supplementary file4 (PDF 481 KB)

Supplementary file5 (PDF 261 KB)

## Data Availability

The datasets generated during and/or analysed during the current study are available from the corresponding author on reasonable request.
