# Supplementary figures and images for: The ERGtools2 package: a toolset for processing and analysing visual electrophysiology data
Source: Doc Ophthalmol. 2025 Apr 12;150(3):169–75. doi: 10.1007/s10633-025-10017-2 (PMC12137496; doi:10.1007/s10633-025-10017-2)

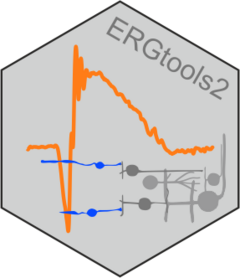

Supplement: Supplementary file 1 — Supplementary file1 (ZIP 622 KB) [file 10633_2025_10017_MOESM1_ESM.zip › S1_ERGtools2_0.8.5/ERGtools2/man/figures/logo.png]

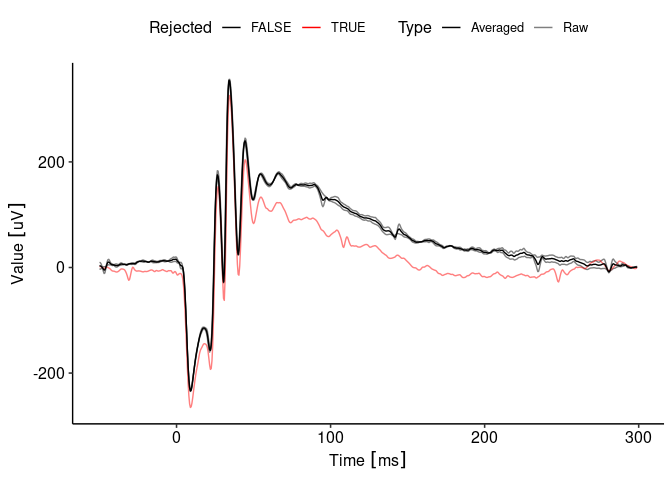

Supplement: Supplementary file 1 — Supplementary file1 (ZIP 622 KB) [file 10633_2025_10017_MOESM1_ESM.zip › S1_ERGtools2_0.8.5/ERGtools2/man/figures/README-example-1.png]

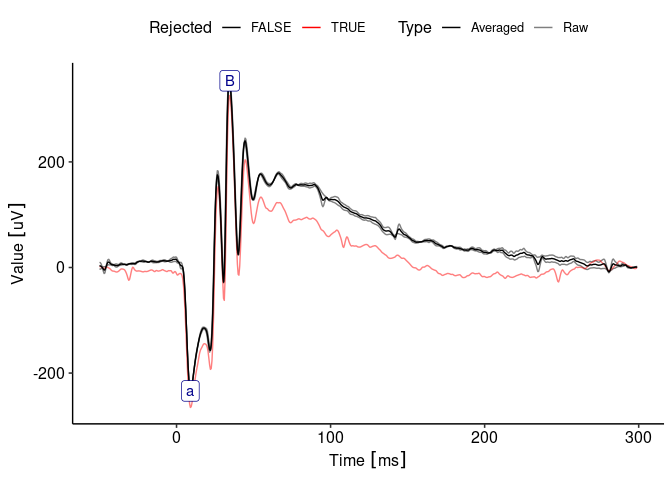

Supplement: Supplementary file 1 — Supplementary file1 (ZIP 622 KB) [file 10633_2025_10017_MOESM1_ESM.zip › S1_ERGtools2_0.8.5/ERGtools2/man/figures/README-example-2.png]

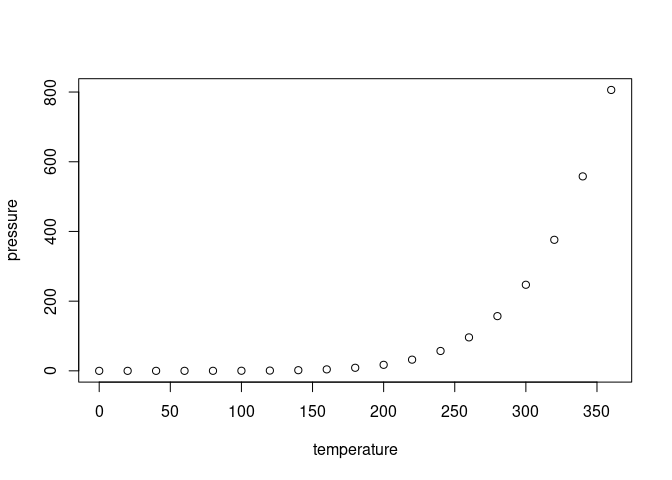

Supplement: Supplementary file 1 — Supplementary file1 (ZIP 622 KB) [file 10633_2025_10017_MOESM1_ESM.zip › S1_ERGtools2_0.8.5/ERGtools2/man/figures/README-pressure-1.png]
