# Supplementary material for: The ERGtools2 package: a toolset for processing and analysing visual electrophysiology data
Source: Doc Ophthalmol. 2025 Apr 12;150(3):169–75. doi: 10.1007/s10633-025-10017-2 (PMC12137496; doi:10.1007/s10633-025-10017-2)
Supplement: Supplementary file 4 — Supplementary file4 (PDF 481 KB) [file 10633_2025_10017_MOESM4_ESM.pdf]

Supplementary methods file

Animal studies

All procedures were performed with the approval of the Giessen Regional Animal Health Authority (File No: G61/2021) and in accordance with the Association for Research in Vision and Ophthalmology (ARVO) Statement for the Use of Animals in Ophthalmic and Vision Research. C57BL/6J mice were purchased from Charles River (Sulzfeld, Germany) and were kept under a 12 h light/dark cycle, with no restriction on food and water. Electroretinogram recordings were performed at Zeitgeber time 3-6 h under general anaesthesia. General anaesthesia was induced and maintained with isoflurane (Baxter, Deerfield, IL, USA). The maintenance dose was approximately 1 % isoflurane in 0.2-0.5 L/min O_2. Anaesthesia was administered using a Univentor 410-Q vaporizer (UNO Roestvaststaal BV, Zevenaar, The Netherlands). Pupil dilation was achieved through local application of Tropicamide (1 %) and Phenylephrine (2.5 %) eye drops. Electroretinograms were recorded using the Celeris Rodent ERG system (Diagnosys LLC, Lowell, MA, USA) employing an integrated light guide stimulator and electrode (D431-10). Oxybuprocaine hydrochloride (4 mg/ml) was instilled into the eye before placement of the stimulator/electrodes.

Electrophysiological Recordings

To demonstrate the functionality of ERGtools2, electroretinogram data recorded from the left eyes of five C57Bl/6J mice (median age 7.4 weeks, interquartile range: 7.1 – 9 weeks) were used. Electroretinograms were recorded using Espion software (Diagnosys) and digitized with a sampling rate of 2000 Hz. Mice were dark-adapted for at least 20 min before recording and were thereafter handled only under dim red light. Before beginning the light-adapted recordings, mice were exposed to 30 cd/m^2 for at least 8 min. The maximum flash stimulus duration was 4 ms.

Recordings were exported from the Espion database into CSV files using the software's inbuilt function. Export settings were set as follows: Export: Table of Contents, Header Table, Marker Table, Stimulus Table and Data Table, Separator: Tab; Options: Titles, Vertical; Include: Steps, Channels, Results; Data Columns: Contents, Results, Sweeps.

Structure and nomenclature of (visual) electrophysiological data

In electrophysiological tests, the fundamental unit of data is the time series, where data traces are recorded over a specified time period in response to a particular stimulus. These recordings

are often repeated multiple times to enhance the signal-to-noise ratio through subsequent averaging. These repetitions are referred to as *Trials,* while a complete set of trials is considered a *Recording*. Recordings may be gathered from different electrodes, often simultaneously, and they are commonly referred to as *Channels*. Sequential recordings conducted under varying stimulus conditions are each referred to as *Steps*. Each Step is characterized by specific stimulus parameters, such as intensity or duration.

The ERGExam object

The structure of the ERGExam object is summarized in **Supplementary Figure 1** (below). Within the object, a single Recording (including all its Trials) is stored in an EPhysData object alongside a time trace. The data slot of ERGExam represents a list of EPhysData objects thus containing all original data from an individual examination. This list of data is associated with a metadata table that contains relevant contextual information, such as the recorded Eye, Channel, and Step ID. Each Step is further defined by the Stimulus Table, which may include a human-readable description of the stimulus along with stimulus intensity, adaptation state, background light intensity, and other parameters. Additional information can be stored in both, the metadata and the stimulus table in user defined columns. Thus, the object contains the data together with all relevant information regarding recording conditions.

ERG and VEP data can be analysed by measuring and comparing the amplitudes of the traces at defined positions, such as the a-wave, which represents the strong initial negative deflection of a recording. These measurements are stored in the Measurements slot. It contains the definition of the individual measurement point, termed *Markers*, including their names, and whether amplitudes for that Marker should be measured relative to any other Marker or with reference to the baseline. It also stores the information on the recording an individual Marker has been placed as well as the exact position on the time axis of that Marker. The amplitudes at the position of the Marker are not stored in the object but are computed each time the measurements are accessed to ensure data consistency.

The EPhysData object additionally contains a slot termed "*StimulusTrace*". This slot can be used (optionally) to store a vector representing the underlying stimulus trace, or any other trace that has the same timing as the actual data, e.g. the readings of a feedback-photodiode. ERGtools2, however, currently does not provide an option to visualize this trace alongside the data.

Supplementary Figures

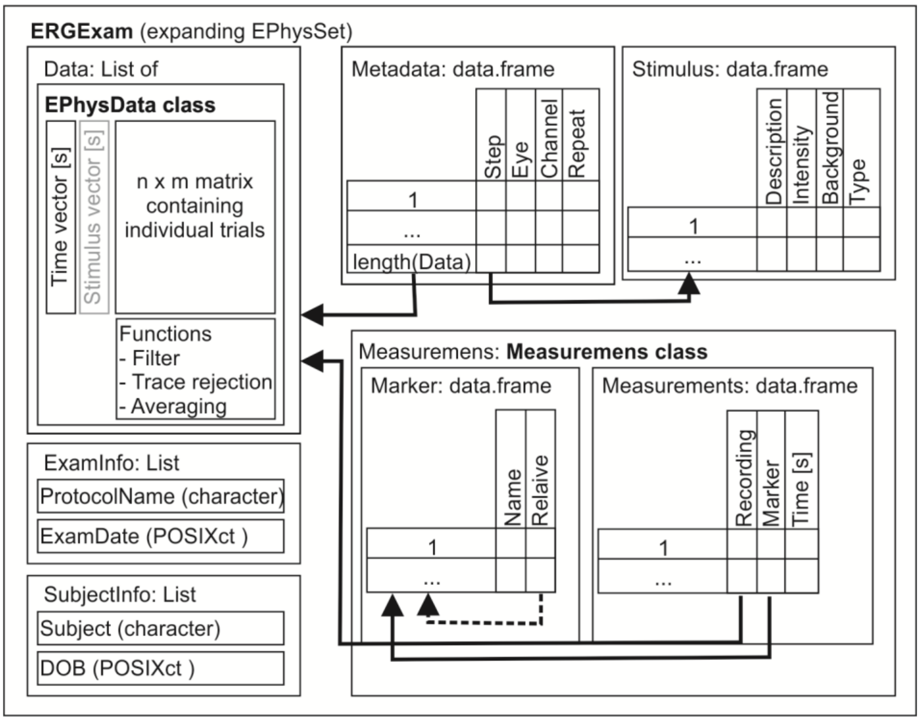

Supplementary Figure 1: The structure of an ERGExam object, the container of visual electrophysiology data in ERGtools2. Details can be found in the supplementary methods file as well as in R typing *“?ERGtools2::`ERGtools2-package`”.*
