# Supplementary material for: The ERGtools2 package: a toolset for processing and analysing visual electrophysiology data
Source: Doc Ophthalmol. 2025 Apr 12;150(3):169–75. doi: 10.1007/s10633-025-10017-2 (PMC12137496; doi:10.1007/s10633-025-10017-2)
Supplement: Supplementary file 5 — Supplementary file5 (PDF 261 KB) [file 10633_2025_10017_MOESM5_ESM.pdf]

Standard Workflow

Importing data

The first step involves creating an EPhysData object that stores the recorded data along with its associated time trace. Data recorded from a single channel of one single eye will be stored into one such object. A new object will be created for the data from the next channel and eye, and so on. This can be done using the *newEPhysData()* function. Once the individual recordings are imported as EPhysData objects, they are assembled into an ERGExam object using the *newERGExam()* function. In the ERGExam object, the individual recordings are stored together with their descriptive metadata such as the recording laterality (e.g., left or right eye) and the channel type (e.g., ERG or VEP). Information defining the stimuli used (e.g., adaptation state and intensity) as well as information on the subject, experimental cohort, and exam date, is also stored in that object.

In practical terms, users first create a list of all their EPhysData objects, this list is then passed to the *newERGExam()* method as the parameter *"Data"*. Metadata and stimulus information are passed on as the parameter "*Metadata*" and "*Stimulus*", respectively. Each entry in the *Metadata* must align with the corresponding entry in *Data*, so it is crucial to ensure that both are ordered correctly. The stimulus information is matched to the *Data* via the Step column, which is an essential part of the *Metadata* table. Additionally, the user provides exam-related information (parameter: *ExamInfo*) in the form of a list containing at least the acquisition protocol name (*ProtocolName*) and the exam date (*ExamDate*). Subject details are provided through the *SubjectInfo* parameter, which is also a list that includes at least the subject's name (*Subject*) and date of birth (*DOB*). A visual summary of the structure of the ERGExam object is shown in Supplementary Figure 1. Although the initial setup of the import routine may require some effort, once established, it can be reused for subsequent exams stored in the same format. R code illustrating two scenarios of such an import procedure is provided as supplemental material (Supplemental Material 2).

Importantly, the raw data remains unaltered throughout the analysis process, ensuring data integrity. ERGtools2 also contains a convenience function for importing ERG exams from Diagnosys's Espion software. Thus, using *ImportEspion()*, exams can be imported with minimal coding effort.

Configuring the data object for analysis

After importing the recordings, the next step is to prepare the data for analysis. ERGtools2 includes functions for averaging repeated trials, filtering, and removing outliers to improve the signal-to-noise ratio. The *SetStandardFunctions()* function streamlines this process by applying a linear detrending filter to each recording. In case of repeated recordings, it then looks for outliers among those and marks them for exclusion. Finally, the averaging function is set to *mean()*. These default settings work well for most standard applications. On special occasions (e.g., when specific bandpass filtering is desired to extract oscillatory potentials) this can be achieved using *FilterFunction()*. Additionally, ERGtools2 offers several alternative algorithms for identifying and excluding outlier trials, and user-defined functions are also supported.

Importantly, when such functions are applied to an ERGExam object, validity checks are automatically performed to ensure internal consistency. For example, if a filter function is changed for a recording from the left eye, the same change must be applied to the corresponding recording from the right eye as well.

Visualizing and subsetting the data

After preprocessing, the exam can be visualized using the ggERGExam() command, which arranges recordings into panels with eyes displayed in columns and channels or adaptation conditions in rows. The different steps, usually representing increasing stimulus intensities, are colour-coded and plotted within the same panel (Figure 1 B in the main text). The user might now find that not all parts of the exam are of relevance for further analysis. Unnecessary recordings can be removed either at the command-line level using *Subset()* or *DropRecordings(),* or interactively using *exploreERGExam()* (Figure 1 C in the main text).

Placing Markers for Measurement

Next, markers must be positioned on the recordings to measure characteristics such as waveform timing and amplitude. The *AutoPlaceMarkers()* function automatically identifies a- and B-wave positions on flash ERG traces as well as P1-, N1-, and P2- waves for VEP traces. Also, peak detection on flicker ERG traces is supported. *AutoPlaceMarkers()* is optimized for the waveform patterns typically observed in wild-type (C57Bl/6J) mice. Once set, marker positions are displayed by *ggERGExam()*, and a table containing timing and amplitudes for all markers can be retrieved by calling *Measurements()*. If the automatic marker placement does not provide satisfactory results, interactive marker placement can be performed using *interactiveMeasurements()* (Figure 1 D in the main text).

Summarizing and analysing a set of exams

In a research setting, an investigator may want to compare exams acquired from subjects belonging to different experimental cohorts. To achieve this, ERGtools2 contains a set of functions that can handle lists of ERGExam objects e.g. to plot the exams side-by-side (*ggPlotRecordings()*), draw intensity response curves (*ggIntensitySequence(),* Figure 2 in the main text), or simply obtain measurements for specific markers for all recordings in the list (*CollectMeasurements()*). The latter can then be used for any downstream analysis, such as descriptive statistics.
